# From One Cancer to Two [¹⁸F]FES PET/CT Redirected Diagnosis and Therapy in a Metastatic Breast Cancer Patient

**DOI:** 10.1097/RLU.0000000000006386

**Published:** 2026-02-23

**Authors:** Anne-Claire Dupont, Mathieu Farges, Benoit Chapelle, Mathilde Cancel, Maria-Joao Santiago Ribeiro

**Affiliations:** Departments of * Nuclear Pharmacy Tours University Hospital, CHRU Tours; † Pneumology; ‡ Medical Oncology; § Nuclear Medicine, CHRU Tours, Tours, France

**Keywords:** ^18^FES PET, breast cancer, double primary malignancies

## Abstract

A 59-year-old woman with an ulcerated right breast lesion was diagnosed with ER-positive invasive ductal carcinoma. Staging [¹⁸F]FDG PET/CT revealed intense uptake in the breast, axillary nodes, a large hypermetabolic pulmonary mass, mediastinal lymphadenopathies, and bone lesions. Because of this metabolic pattern, [¹⁸F]FES PET/CT was performed, demonstrating [¹⁸F]FES-avid bone metastases but no uptake in the lung mass. Biopsy confirmed a BRAF V600E-mutated lung adenocarcinoma. [¹⁸F]FES PET/CT proved crucial in differentiating synchronous primary malignancies from metastatic spread and guiding targeted therapy with dabrafenib-trametinib.

**FIGURE 1:**
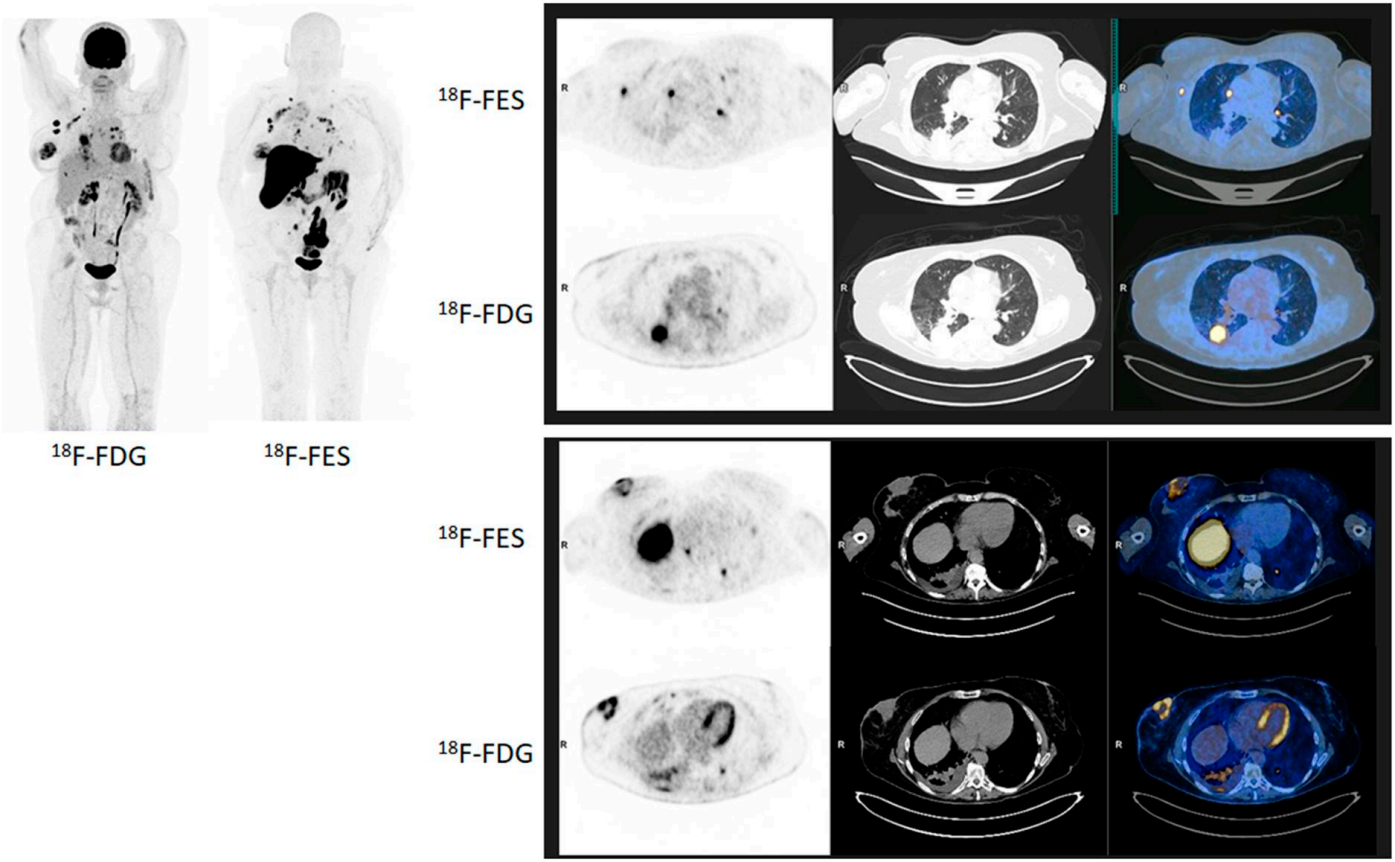
A 59-year-old woman presented with a right breast lesion after local trauma. Clinical examination revealed an ulcerated lesion involving the entire areola and bleeding on contact. Biopsy confirmed an invasive ductal carcinoma grade II, estrogen receptor positive (ER+), progesterone receptor negative (PR–), and HER2 negative (0). A staging [¹⁸F]FDG PET/CT demonstrated intense hypermetabolism of the right breast lesion, multiple hypermetabolic axillary lymph nodes, a large right lower lobe pulmonary mass, mediastino-hilar lymphadenopathies, multiple nodular lesions, and an osteolytic lesion in the left sacral wing. The lung lesion also shows intense uptake of [¹⁸F]FDG, although to a slightly lesser degree than the breast lesion. The pulmonary lesion was large and demonstrated intense [¹⁸F]FDG uptake, raising suspicion for a second primary malignancy rather than a metastasis. Given this metabolic pattern, a 16α-[¹⁸F]fluoro-17β-estradiol ([¹⁸F]FES) PET/CT was subsequently performed to assess estrogen receptor expression and further characterize potential metastatic disease. The scan revealed a dissociated uptake pattern, with no [¹⁸F]FES uptake in the right lower lobe pulmonary mass or mediastino-hilar lymphadenopathies, whereas the sacral and iliac bone lesions demonstrated FES uptake, consistent with ER-positive breast cancer metastases. CT-guided biopsy of the pulmonary lesion confirmed a primary bronchopulmonary adenocarcinoma, ALK-negative and ROS1-negative, PD-L1 20%, harboring BRAF V600E and PIK3CA mutations. These findings established the coexistence of 2 synchronous metastatic primaries: an ER-positive breast carcinoma and a BRAF-mutated lung adenocarcinoma. Targeted therapy with dabrafenib and trametinib was initiated for the metastatic lung adenocarcinoma, whereas hormonal therapy with an aromatase inhibitor was continued for the breast cancer. At the 3-month follow-up, CT imaging showed a partial response, with regression of the pulmonary lesion, mediastinal lymph nodes, and carcinomatous lymphangitis, whereas the right breast mass remained stable. Clinical tolerance to therapy was good. Double primary malignancies involving the breast and lung are well-documented in the medical literature.^1^ The diagnostic value of [¹⁸F]FDG PET/CT in the staging and management of breast cancer is well established,^2^ as is that of [¹⁸F]FES PET/CT for assessing estrogen receptor expression.^3–7^ However, reports describing synchronous breast and lung primaries evaluated with both radiotracers remain extremely rare. To our knowledge, only one case, reported by Lee et al,^8^ described a similar presentation but without available [¹⁸F]FDG imaging. In conclusion, [¹⁸F]FES PET/CT played a pivotal role in differentiating synchronous primary malignancies from metastatic dissemination, enabling accurate molecular characterization and optimization of therapeutic management in this complex oncologic setting.
